# Aluminium Binding to Modified Amyloid-β Peptides: Implications for Alzheimer’s Disease

**DOI:** 10.3390/molecules25194536

**Published:** 2020-10-03

**Authors:** Cosmin Stefan Mocanu, Monica Jureschi, Gabi Drochioiu

**Affiliations:** Faculty of Chemistry, “Al. I. Cuza” University of Iasi, 11 Carol I, 70605 Iasi, Romania; cosmin.mocanu@chem.uaic.ro (C.S.M.); jureschi.monica@yahoo.com (M.J.)

**Keywords:** amyloid-β peptides, modified Aβ peptide fragments, aluminium ions, metal binding, mass spectrometry, circular dichroism spectroscopy, atomic force microscopy, FT-IR, Alzheimer’s disease

## Abstract

Aluminium (Al) is clearly neurotoxic and considerable evidence exists that Al may play a role in the aetiology or pathogenesis of Alzheimer’s disease (AD). Nevertheless, the link between AD pathology and Al is still open to debate. Therefore, we investigated here the interaction of aluminium ions with two Aβ peptide fragments and their analogues. First, we synthesised by the Fmoc/*t*Bu solid-phase peptide synthesis (SPPS) strategy using an automated peptide synthesiser two new peptides starting from the Aβ_(1–16)_ native peptide fragment. For this purpose, the three histidine residues (H^6^, H^13^, and H^14^) of the Aβ_(1–16)_ peptide were replaced by three alanine and three serine residues to form the modified peptides Aβ_(1–16)_A_3_^6,13,14^ and Aβ_(1–16)_S_3_^6,13,14^ (primary structures: H-^1^DAEFR**A**DSGYEV**AA**QK^16^-NH_2_ and H-^1^DAEFR**S**DSGYEV**SS**QK^16^-NH_2_). In addition, the Aβ_(9–16)_ peptide fragment (H-^9^GYEVHHQK^16^-NH_2_) and its glycine analogues, namely Aβ_(9–16)_G_1_^10^, (H-^9^G**G**EVHHQK^16^-NH_2_), Aβ_(9–16)_G_2_^13,14^ (H-^9^GYEV**GG**QK^16^-NH_2_), and Aβ_(9–16)_G_3_^10,13,14^ (H-^9^G**G**EV**GG**QK^16^-NH_2_), were manually synthesised in order to study Al binding to more specific amino acid residues. Both the peptides and the corresponding complexes with aluminium were comparatively investigated by mass spectrometry (MS), circular dichroism spectroscopy (CD), atomic force microscopy (AFM), scanning electron microscopy (SEM), and Fourier transform infrared spectroscopy (FT-IR). Al–peptide molecular ions and Al-fragment ions were unambiguously identified in the MS and MS/MS spectra. AFM images showed dramatic changes in the film morphology of peptides upon Al binding. Our findings from the investigation of N-terminal 1-16 and even 9-16 normal and modified sequences of Aβ peptides suggest that they have the capability to be involved in aluminium ion binding associated with AD.

## 1. Introduction

The prevalence of Alzheimer’s disease (AD) continues to increase worldwide, becoming a great healthcare challenge of the twenty-first century [1]. AD is a progressive neurodegenerative disorder that gradually deprives the patient of cognitive function, being characterised by the presence of senile plaques and neurofibrillary tangles in the hippocampus and neocortex of the brain [2]. Intracranial amyloid aggregates, which are dependent on the proteolysis of the Aβ precursor protein (APP), are usually found in AD brains [3,4]. Such aggregates are made up of amyloid-β (Aβ) peptides, cholesterol, and metal ions and generate so-called neural plaques. The proteolytic Aβ peptides are normally degraded and removed, but they may accumulate in AD brains, mainly in presynaptic terminals of neurons to form insoluble plaques [5]. Peptide aggregates lead to the death of affected hippocampal neurons [6]. Aβ plaques contribute to neuronal loss and ultimate failure of cognitive function [7]. The interaction of Aβ peptides with ions of copper, zinc, iron, aluminium, manganese, or mercury may contribute to neuronal damage [8,9,10]. Once Aβ begins to accumulate, it then promotes the build-up of Tau [11]. Hence, blocking the production and aggregation of Aβ peptides is of interest in AD therapy [12]. Aβ aggregation, a known contributor in AD pathogenesis, is triggered by several metal ions through occupational exposure and disrupted metal ion homeostasis [13]. Thus, amyloid-β aggregates are associated with AD and can be promoted by traces of metal ions such as aluminium, iron, zinc, copper, manganese, lead, cadmium, or mercury [14]. It is well known that these elements cause conformational changes of Alzheimer’s amyloid-β peptides [15]. Although Al is a neurotoxic agent, the link of Al to the aetiology of various serious neurological disorders such as Alzheimer’s disease (AD) remains controversial [16,17]. Nevertheless, Al has been included among the potential risk factors for the aetiology of AD [15,18]. Al may also accelerate the proteolytic processing of APP by suppression of the inhibitor domain in the Alzheimer’s brain [19]. Moreover, Al induces the accumulation of Aβ peptides and tau protein in the brains of experimental animals [20]. As a result, the Aβ peptide may accumulate and initiate plaque formation. There is evidence that Al and Aβ are co-located in a senile plaque-like structure [21]. However, a natural phenolic antioxidant, curcumin, is capable of binding with Al ions, and the metal–curcumin complex can inhibit the transition from less structured oligomers to β-sheet-rich protofibrils [22].

Recently, multiple microsecond-long molecular dynamics simulations of complexes of amyloid-β peptides with aluminium ions have also been reported [23]. On the other hand, in order to highlight the importance of each amino acid residue from the Aβ region involved in the formation of complexes with amyloidogenic substances, peptides containing alanine single-site mutations within the Aβ binding sequence can be produced by solid-phase peptide synthesis, whereas the complex formation could be investigated for each mutant peptide by mass spectrometry (MS) [24]. Mass spectrometric investigation of non-covalent complex formation between amyloid-β peptides and various toxic substances tested for their impact in AD is an important step for clarifying their mechanism of action [25]. Experiments based on MS, infrared (IR), UV–vis and microscopic techniques revealed significant changes in the structure of peptides, including their aggregation and fibrillation, associated with metal binding [26,27,28,29]. We have recently been looking for evidence of such complexity using MS and other bioanalytical techniques [30,31,32].

Very little is also known about how pH and time-dependent metal ion binding to specific Aβ sequences influence the structure, aggregation, fibrillation, and plaque formation in vitro and in AD brains [33,34]. Therefore, we aimed in this study to address this knowledge gap and determine the relative contribution of aluminium binding of several Aβ analogue peptides. Towards this goal, we used a combination of MS, circular dichroism spectroscopy (CD), atomic force microscopy (AFM), SEM, FT-IR, and other techniques to establish the role played by the three histidine residues of the Aβ_(1–16)_ sequence in binding aluminium ions, if any. We followed the hypothesis that replacing the histidine residues in positions 6, 13, and 14 of Aβ_(1–16)_ peptide fragment sequence H-^1^DAEFR**H**DSGYEV**HH**QK^16^-NH_2_ with alanine [24] or serine may result in two new variants of Aβ(_(1–16)_ peptide that may afford a different pattern of interaction with Al ions. Consequently, the modified peptides Aβ_(1–16)_A_3_^6,13,14^ (H-^1^DAEFR**A**DSGYEV**AA**QK^16^-NH_2_) and Aβ_(1–16)_S_3_^6,13,14^ (H-^1^DAEFR**S**DSGYEV**SS**QK^16^-NH_2_) were synthesised by the Fmoc/*t*Bu solid-phase peptide synthesis (SPPS) strategy and purified by reverse phase-high performance liquid chromatography (RP-HPLC). Furthermore, because the amino acid residues Glu^3^, Asp^7^, and Glu^11^ are suspected to bind Al [23], we decided to use in our experiments the smaller peptide Aβ_(9–16)_ and several analogues, which do not contain the first two amino acid residues in their sequences. Consequently, in the sequence of the Aβ_(9–16)_ peptide fragment (GYEVHHQK), we replaced either the histidine residues or the tyrosine one with glycine to form new molecules of more flexible peptides, namely Aβ_(9–16)_G_1_^10^, (G**G**EVHHQK), Aβ_(9–16)_G_2_^13,14^ (GYEV**GG**QK), and Aβ_(9–16)_G_3_^10,13,14^ (G**G**EV**GG**QK). Finally, matrix-assisted laser desorption ionization time-of-flight (MALDI-ToF) mass spectrometry, including MS/MS study, and Fourier transform infrared (FT-IR) spectroscopy revealed an unexpected pattern of aluminium ion binding to both Aβ_(1–16)_ and Aβ_(9–16)_ peptides and its analogues.

## 2. Results

### 2.1. Peptide Synthesis

First, two modified peptides, analogues of the Aβ_(1–16)_ peptide fragment, were synthesised by SPPS according to the Fmoc/*t*-Bu strategy on an automated peptide synthesiser. In the native Aβ_(1–16)_ peptide, which was also synthesised under the same conditions, the three histidine residues were replaced with alanine (Aβ_(1–16)_A_3_^6,13,14^:H-^1^DAEFR**A**DSGYEV**AA**QK^16^-NH_2_) and serine (Aβ_(1–16)_S_3_^6,13,14^:H-^1^DAEFR**S**DSGYEV**SS**QK^16^-NH_2_), in order to produce two peptides lacking the imidazole ring but with similar flexibility to the native Aβ_(1–16)_ peptide. The serine-rich peptide also contains more hydroxyl groups than the native peptide and could bind better aluminium ions. In addition, hydroxyl groups can induce the formation of hydrogen bonds that could lead to the aggregation and fibrillation of free serine peptides.

**Figure 1:**
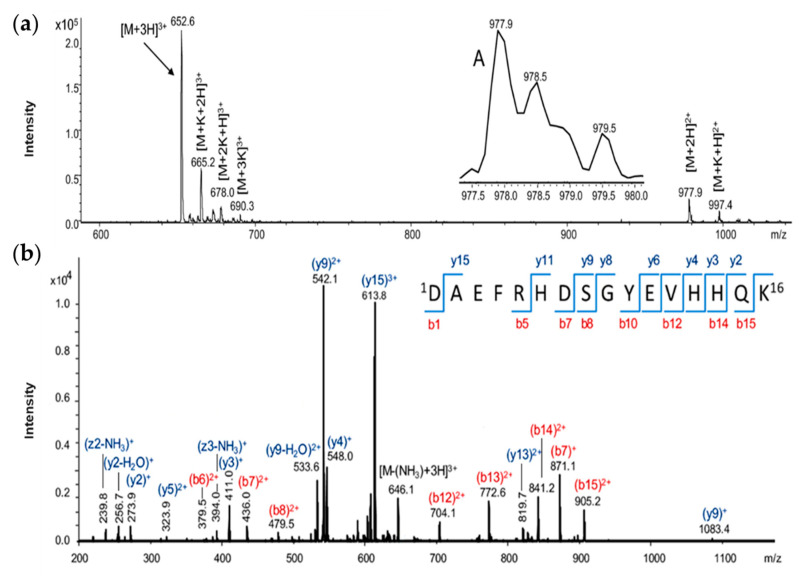
(**a**) Electrospray ionisation mass spectrometry (ESI-MS) analysis of Aβ_(1–16)_; (**b**) MS/MS spectrum of [M + 3H]^3+^ molecular ion.

Second, the Aβ_(9–16)_ peptide fragment and its three analogues, namely Aβ_(9–16)_G_1_^10^ (H-^9^G**G**EVHHQK^16^-NH_2_), Aβ_(9–16)_G_2_^13,14^ (H-^9^GYEV**GG**QK^16^-NH_2_), and Aβ_(9–16)_G_3_^10,13,14^ (H-^9^G**G**EV**GG**QK^16^-NH_2_), were manually synthesised on a Rink amide resin as solid support using the same fluorenylmetoxycarbonyl chemistry. The resulting crude peptides were purified by RP-HPLC chromatography on a C18 column. Here, Figure 1 refers only to the characterisation with an electrospray ionisation (ESI) ion trap mass spectrometer of the Aβ_(1–16)_ peptide, but all the other peptides used in our experiments were measured by the same procedure (not shown).

In the ESI ion trap mass spectrum of the Aβ_(1–16)_ peptide (Figure 1a), the most intense signal observed at 652.1 *m*/*z* was attributed to the triple-charged ion [M + 3H]^3+^, while the low-intensity signal from 977.3 *m*/*z* was assigned to the double-charged ion [M + 2H]^2+^. In addition, double-charged ions of potassium adduct [M + K + H]^2+^ were noticed at *m*/*z* 997.4 and the triple-charged one [M + K + 2H]^3+^ was present at *m*/*z* 665.2 in the mass spectrum. Other potassium-containing molecular ions such as [M + 2K + H]^3+^ and [M + 3K]^3+^ were found at *m*/*z* 678.0 and *m*/*z* 690.3. While a simple mass spectrometer determines the mass of a molecular ion based on the mass/electric charge ratio, tandem mass spectrometry (MS/MS) is used to select a particular molecular ion to determine the mass of the resulting fragment ions and a certain fragmentation pattern. If a molecular ion of a metal complex is fragmented, the fragment containing the metal ion can be stabilised and coordination sites can be determined by collision-induced dissociation (CID). The structure of Aβ_(1–16)_ was also investigated by tandem mass spectrometry (MS/MS), based on a collision-induced dissociation process [32]. The most intense peak in the mass spectrum, belonging to the [M + 3H]^3+^ ion, was selected and subjected to MS/MS fragmentation (Figure 1b). The two intense peaks from *m*/*z* 542.1 and *m*/*z* 613.8 were assigned to the *y*-type fragments (y_9_^2+^ and y_15_^3+^). This type of fragmentation indicated the reduced stability of the peptide bond between the aspartic acid and alanine as well as serine residues. The presence of such peaks in the MS/MS spectra of peptides and their modification in the spectra of aluminium complexes of the investigated peptides may suggest not only the binding site of the metal ion, but also its influence upon the fragmentation mechanism in the CID process. Obviously, the MS/MS spectrum of Aβ_(1–16)_ peptides contains mono-charged ions such as y_2_^+^ (at *m*/*z* 273.9), y_3_^+^ (*m*/*z* 411.0), b_7_^+^ (*m*/*z* 871.1), or y_9_^+^ (*m*/*z* 1083.4). Among the double-positively charged fragments, fragments like y_5_^2+^ (*m*/*z* 323.9), b_6_^2+^ (*m*/*z* 379.5), b_7_^2+^ (*m*/*z* 436.0), b_8_^2+^ (*m*/*z* 479.5), b_12_^2+^ (*m*/*z* 704.1), b_13_^2+^ (*m*/*z* 772.6), b_14_^2+^ (*m*/*z* 841.2), and b_15_^2+^ (*m*/*z* 905.2) were identified. Some unusual fragments also appeared in the mass spectrum, such as [y_9_-H_2_O]^2+^ at *m*/*z* 533.6, resulting from water removal or ammonia at *m*/*z* 646.1 for the [M-(NH_3_)+3H]^3+^ ion, *m*/*z* 239.8 (z2-NH_3_)^+^, and another at *m*/*z* 394 attributed to the (z_3_-NH_3_)^+^ ion. The unusual peaks were specific to the amidated C-terminal, the -CONH_2_ group being prone to dehydration by the loss of a water molecule and the formation of a -CN functional group.

**Figure 2:**
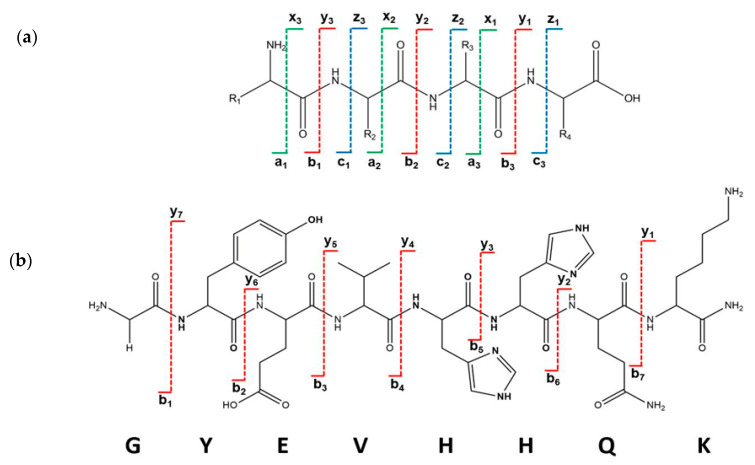
(**a**) Possible ion fragments which result in the collision-induced dissociation of the Aβ_(9–16)_ peptide, of which some may appear in the MS/MS spectra; (**b**) the structure of *b*- and *y*-type fragment ions of the GYEVHHQK-NH_2_ peptide.

**Figure 3:**
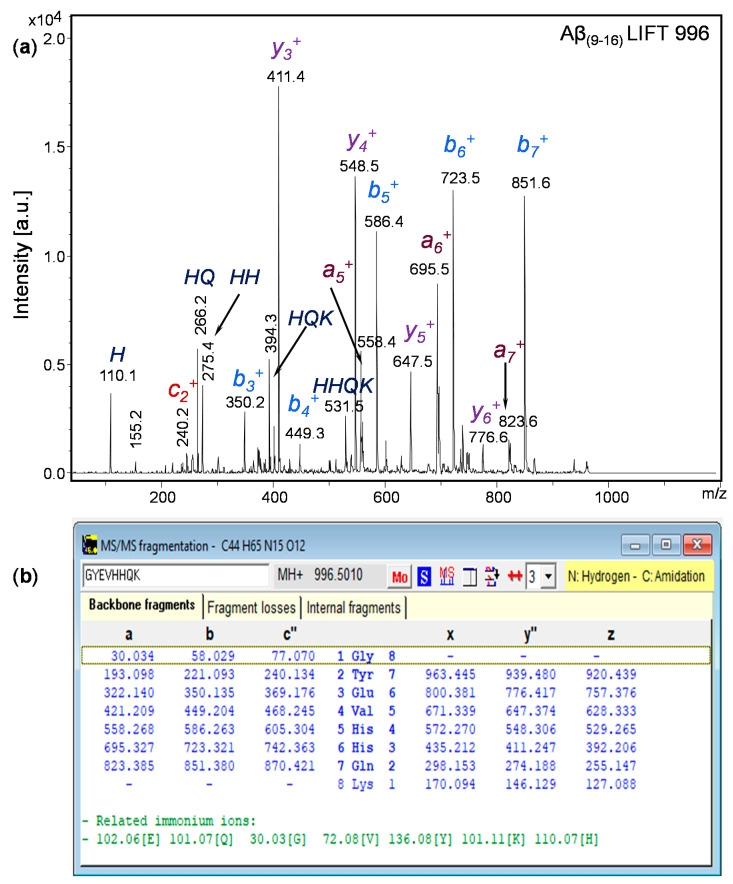
MS/MS fragmentation of free Aβ_(9–16)_ peptide: (**a**) MS/MS spectrum of amidated Aβ_(9–16)_ peptide; (**b**) theoretical calculation using GPMAW software of *m*/*z* values of the resulting fragments within the collision-induced dissociation.

The investigation of the MS/MS spectrum of a shorter peptide such as the Aβ_(9–16)_ peptide fragment revealed a wealth of information about its structure. Typical ion fragments that may result from collision-induced dissociation are exemplified in Figure 2. The MS/MS spectrum in Figure 3 shows that the fragmentation occurred mainly in histidine residues; the highest peak was attributed to the y_3_^+^ fragment resulting from the division of the two histidine residues. In addition, the y_4_^+^ fragment visible at *m*/*z* 548.5 showed a rupture of the peptide bond between histidine and valine. However, while the presence of a b_5_^+^ fragment ion confirms that the peptide bond between the two histidine residues can be broken, the relatively intense peaks of the b_6_^+^ and b_7_^+^ fragment ions may explain the formation of HQ and HH fragments. The *m*/*z* values calculated with GPMAW software confirmed both the structure and the stability of the peptide molecule.

### 2.2. CD Spectroscopy of Aβ_(9–16)_ Peptide Fragment

**Figure 4:**
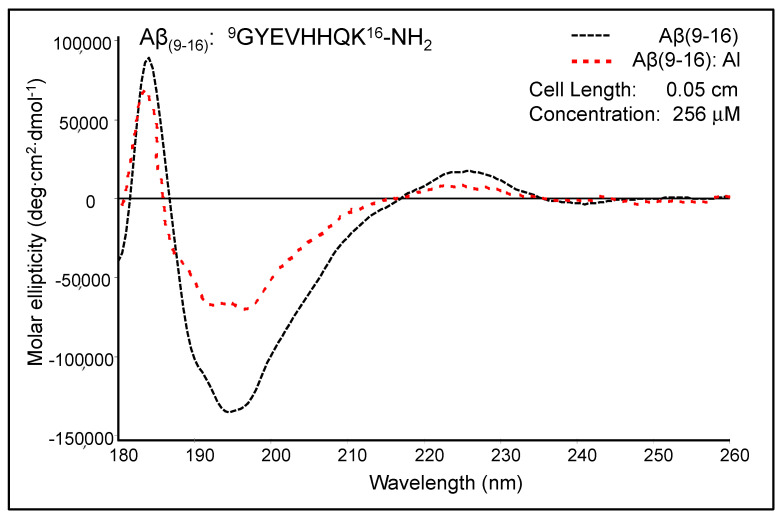
The effect of aluminium ions on the circular dichroism (CD) spectrum of Aβ_(9–16)_ peptide (pH 7.4; (1:1 Al:Aβ_(9–16)_ peptide molar ratio).

We started several CD experiments a long time ago to prove conformational changes of peptides upon metal binding. Figure 4 shows the moderately modified CD spectrum of Aβ_(9–16)_ upon the addition of Al^3+^ ions at pH 7.4. The free Aβ_(9–16)_ peptide showed a CD spectrum characterised by two positive absorption bands at 184 nm (89,384 deg·cm^2^·dmole^−1^) and 226 nm (17,214 deg cm^2^ dmole^−1^), as well as a negative absorption band at 193–197 nm (132,353 deg cm^2^ dmole^−1^). The shape of the Aβ_(9–16)_ CD spectrum suggested rather a random coil conformation of this small peptide, especially due to the presence of a minimum at about 197 nm. In addition, the solution of pure Aβ_(9–16)_ may have a significant proportion of α-helical conformers, as suggested by the positive maximum at 184 nm. However, a full helical protein has a maximum shifted to 191–193 nm in the CD spectrum. The profile of the Aβ_(9–16)_ far-UV CD spectrum was much changed in the presence of Al^3+^ ions at a 1:1 molar ratio. We thus suspected a certain increase in the proportion of β-turn populations following the addition of Al^3+^ ions. Besides, the spectrum appears to have characteristics closer to those of an α-helix-like spectrum. Nevertheless, the strong double minimum was found at 191.5 nm (65,138 deg cm^2^ dmole^−1^) and 197.5 nm (67,925 deg cm^2^ dmole^−1^), and not at 208 and 222 nm, as the case of total α-helical conformers. On using Dicroprot software of the CD instrument, the free peptide had the following proportions of conformers: 0% α-helix, 25.66% β-sheet, 22.23% β-turn, and 52.11% random coil. Upon aluminium addition, these proportions slightly changed (3.34% α-helix, 24.88% β-sheet, 22.84% β-turn, and 48.94% random coil).

We thus hypothesised that because the samples of Aβ_(9–16)_ were dissolved in an ammonium acetate buffer (15 mM) at pH 7.4, the aluminium ions bound very weakly to the peptide backbone and, as a result, the peptide conformation was only slightly altered. Therefore, we designed new experiments with Aβ_(9–16)_ and its analogues at a lower pH value (pH 6.6). 

### 2.3. Interaction of Aβ_(9–16)_ Peptide and Its Analogues with Al Ions at pH 6.6 

The aluminium may have had a higher affinity to amyloid peptides at a lower pH due to the reaction of this metal with acidic residues in the peptide sequences. Here, the existence of Al–peptide complexes was highlighted by MS measurements. The experimental values were then compared with those calculated theoretically. Mass spectra unequivocally showed the formation of aluminium complexes of Aβ_(9–16)_ peptides of the formula [M + Al − 2H]^+^, where M is Aβ_(9–16)_, Aβ_(9–16)_G_1_^10^, Aβ_(9–16)_ G_2_^13,14^, and Aβ_(9–16)_G_3_^10,13,14^. It is thought that the binding capacity of aluminium ions is closely related to the toxicity of amyloid peptides, with high concentrations of aluminium being found in senile plaques [35]. Thus, determining how the metal ion binds to the amyloid fragment, together with the study of conformational changes undergone by peptides in the presence of aluminium, could provide important information for understanding AD pathology. Here, both the Aβ_(9–16)_ fragment and its analogues produced by replacing tyrosine and histidine residues with glycine (Aβ_(9–16)_G_1_^10^, Aβ_(9–16)_ G_2_^13,14^, and Aβ_(9–16)_G_3_^10,13,14^) were analysed by MALDI-ToF mass spectrometry in the absence and in the presence of aluminium ions.

**Figure 5:**
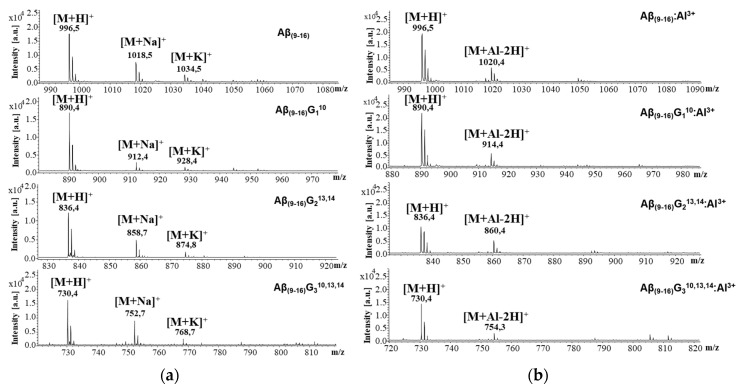
Matrix-assisted laser desorption ionization time-of-flight mass spectrometry **(**MALDI-ToF MS) analysis of (**a**) Aβ_(9–16)_ peptide (GYEVHHQK-NH_2_) and its glycine analogues: Aβ_(9–16)_G_1_^10^, (G**G**EVHHQK-NH_2_), Aβ_(9–16)_G_2_^13,14^ (GYEV**GG**QK-NH_2_), and Aβ_(9–16)_G_3_^10,13,14^ (G**G**EV**GG**QK-NH_2_); (**b**) MALDI-ToF MS spectra of Aβ_(9–16)_ peptides in the presence of aluminium ions (AlCl_3_).

Figure 5a presents the mass spectra of peptides recorded in the absence of aluminium ions, while the interaction with metal ions can be noticed in Figure 5b. The juxtaposition of these spectra allows for the assessment of interactions between the studied peptides and aluminium ions. The free peptides generated several peaks attributed to the molecular ions of the protonated peptide [M + H]^+^, as well as to the adducts with sodium [M + Na]^+^ and potassium [M + K]^+^. The presence of aluminium ions led to the formation of a peptide–metal complex that generated molecular ions of the [M + Al − 2H]^+^ type. However, the peaks of sodium and potassium adducts of all peptides disappeared in the MS spectrum taken in the presence of aluminium chloride, suggesting that these ions were replaced by Al ions. Although MALDI-ToF MS measurements cannot estimate the proportion of peptide molecules bound to metal ions, the peak intensity of the aluminium complexes suggests that the Aβ_(9–16)_ peptide had a considerable affinity to Al ions (intensity ratio [M + Al − 2H]^+^/[M + H]^+^ = 31.39%). At the same time, the highest intensity ratio was calculated for Aβ_(9–16)_G_2_^13,14^ (50.45%), whereas the lowest was that of the Aβ_(9–16)_G_3_^10,13,14^ peptide (20.68%). These findings suggest that the glycine-induced molecule flexibility could play an important role in Al binding. In addition, they confirm that histidine residues cannot be bound to Al.

**Figure 6:**
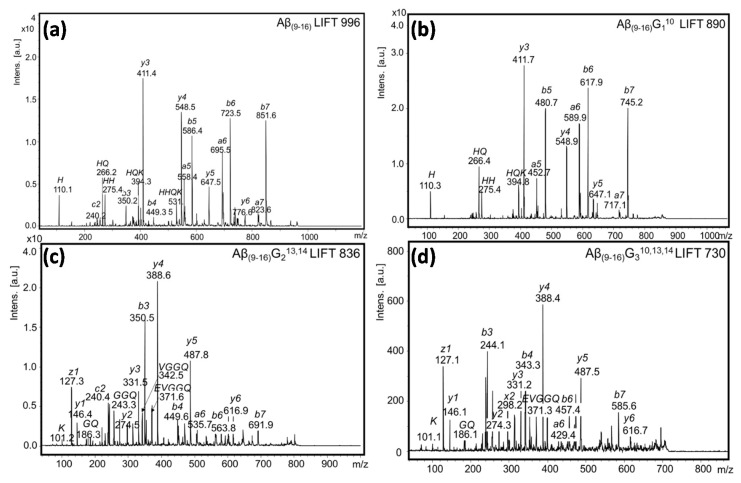
MS/MS spectra of the modified Aβ_(9–16)_ peptides: (**a**) Aβ_(9–16)_; (**b**) Aβ_(9–16)_G_1_^10^; (**c**) Aβ_(9–16)_G_2_^13,14^; (**d**) Aβ_(9–16)_G_3_^10,13,14^.

Before fragmenting the peak of the [M + Al − 2H]^+^ complex, we studied the fragmentation of the peptide molecules in order to compare the resulting ions (Figure 6). The MS/MS spectra showed that the four Aβ_(9–16)_ peptides differ greatly in the type and intensity of the fragments resulting from the CID process. Thus, in the spectrum of Aβ_(9–16)_ peptides and that of tyrosine-free peptides, Aβ_(9–16)_G_1_^10^, the y_3_^+^ fragment (at *m*/*z* 411.4 and 411.7) afforded the most intense peaks, while the fragment y_4_^+^ of glycine-rich peptides (Aβ_(9–16)_G_2_^13,14^ and Aβ_(9–16)_G_3_^10,13,14^) had the highest intensity. In addition, the order of peak intensity was as follows: Aβ_(9–16)_, y_3_^+^ > y_4_^+^ > b_6_^+^ > b_7_^+^; Aβ_(9–16)_G_1_^10^, y_3_^+^ > b_6_^+^ > b_5_^+^> b_7_^+^; Aβ_(9–16)_G_2_^13,14^, y_4_^+^ > b_3_^+^ > y_5_^+^ > z_1_^+^; Aβ_(9–16)_G_3_^10,13,14^, y_4_^+^ > b_3_^+^ > z_1_^+^ > y_5_^+^.

Following MS experiments with the Al–peptide complexes, we selected the parent ion [M + Al − 2H]^+^ and subjected it to CID fragmentation. The assignment of the signals observed in the MS/MS spectra of [M + Al − 2H]^+^ ions was performed by comparing the theoretical molecular masses of the ions resulting from the CID fragmentation with the experimentally recorded values. The experimentally determined *m*/*z* values were in accordance with the theoretical values. A comparison of the theoretical *m*/*z* values estimated using GPMAW software with the experimental data can be seen in Table 1.

**Figure 7:**
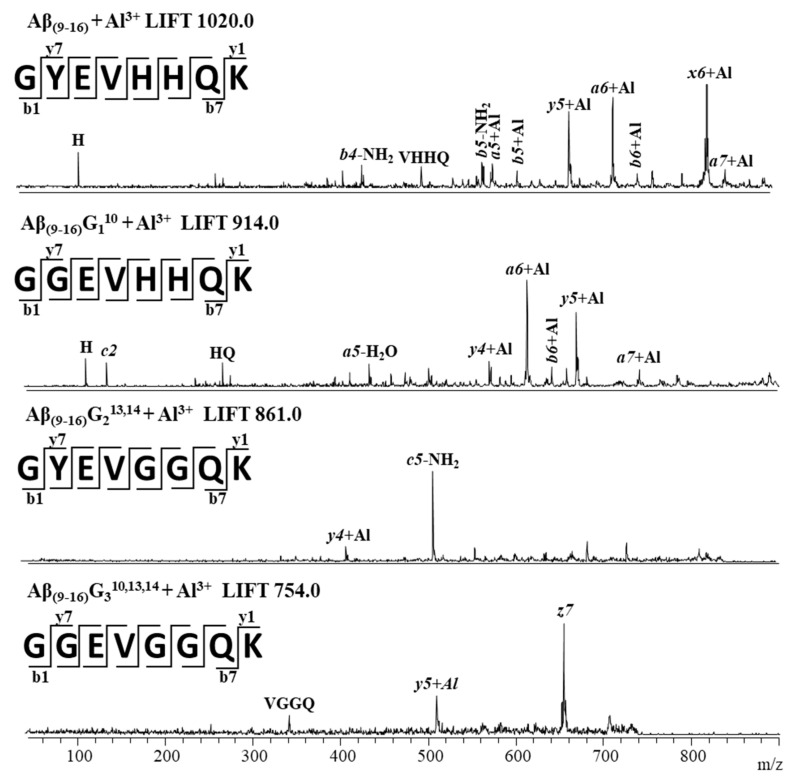
MALDI-ToF tandem (MS/MS) mass spectra of the [M + Al − 2H]^+^ ions of the Aβ_(9–16)_ amyloid peptide fragment and its analogues with glycine. All *m*/*z* values shown are monoisotopic.

Aβ_(9–16)_ peptides, containing two histidine residues in the molecule, generated numerous peaks, most of them bound to Al ions, such as [Al + *x_6_*^+^ − 3H]^+^, [Al + *a_6_*^+^ − 3H]^+^, [Al + *y_5_*^+^ − 3H]^+^, [Al + *a_5_*^+^ − 3H]^+^, and [Al + *b_5_*^+^ − 3H]^+^ (Figure 7). Only a few peaks were assigned to peptide fragment ions without aluminium. The Aβ_(9–16)_G_1_^10^ peptide, obtained by replacing the tyrosine residue with glycine, also had many peaks in the MS/MS spectrum, which were attributed to Al-containing peptide fragment ions such as [Al + *a_6_*^+^ − 3H]^+^, [Al + *y_5_*^+^ − 3H]^+^, [Al + *y_4_*^+^ − 3H]^+^, [Al +*b_6_*^+^ − 3H]^+^, and [Al + *a_7_*^+^ − 3H]^+^, suggesting that aluminium protected the peptide bond between the two histidine residues. In addition, while the fragmentation of the free peptides occurred mainly at the peptide bond between the histidine residues to form the y_3_^+^ fragment, Al induced different fragmentation to generate the *a*-type fragments containing metal ions.

As for the glycine-enriched peptides, only the *y_4_*^+^ and *y_5_*^+^ fragment ions were found to possess Al bound to their backbone, which suggests a weaker interaction of these peptides with Al ions. Besides, the presence of [*b_4_*-NH_2_]^+^ and [*b_5_*-NH_2_]^+^ structures was noticed in the MS/MS spectrum of the Aβ_(9–16)_ peptide and [*c_5_*-NH_2_]^+^ in that of the Aβ_(9–16)_G_2_^13,14^ peptide, which suggests a possible cyclisation followed by removing the NH_2_ radical induced by Al. The main peak in the MS/MS spectrum of the Aβ_(9–16)_G_3_^10,13,14^ peptide was assigned to *z_7_*^+^, while this fragment does not appear in the spectrum of free peptides. This may be evidence that the binding of Al to the investigated peptides changed their stability and induced different fragmentation patterns. Although aluminium ions may be not capable of binding to the histidine residues of Aβ peptides, they can bind easily to HHQK peptide fragments and less well to GYEV or GGEV residues. However, glycine residues in the modified Aβ_(9–16)_ peptides induce a high degree of flexibility, which may inhibit Al binding to peptides.

### 2.4. Fourier Transform Infrared Spectroscopy (FT-IR)

**Figure 8:**
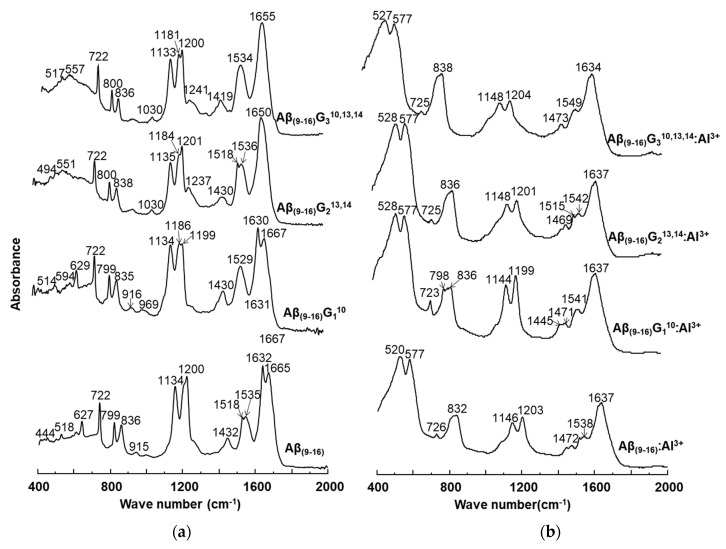
FT-IR spectra of the Aβ_(9–16)_ amyloid peptide and its corresponding analogues that were recorded: (**a**) in the absence of aluminium ions; (**b**) in the presence of aluminium ions.

The conformational changes undergone by the peptides in the presence of aluminium ions were also investigated by FT-IR (Figure 8). Comparison of peptide spectra before (Figure 8a) and after incubation with metal ions (Figure 8b) highlighted the structural influence of aluminium reflected in the changes in absorption values. For instance, important changes were noticed in the range of 1000–2000 cm^−1^, which provide relevant structural information. It was also noted that the 400–1000 cm^−1^ region of the peptide spectrum showed intense absorption characteristic of aluminium ions bound to oxygen or nitrogen atoms (Figure 8b).

The FT-IR spectra of the four peptides showed characteristic bands for the individual peptides, but each of them exhibited a relatively high peak at 722 cm^−1^. If the Aβ_(9–16)_ and Aβ_(9–16)_G_1_^10^ peptides had absorption bands at 627 cm^−1^ and 629 cm^−1^, respectively, these peaks disappeared from the FT-IR spectra of glycine-rich peptides, probably due to the removal of the imidazole ring. Upon adding Al, the 400–725 cm^−1^ region changed dramatically. Two large and high-intensity bands appeared due to Al-O and Al-N bonds, one of them at 520–528 cm^−1^ and the other one at 577 cm^−1^. Another large band appeared at 832-838 cm^−1^, being generated from the two narrow peaks 799–800 cm^−1^ and 835–838 cm^−1^. The presence of aluminium ions also determined a decrease in the intensity of the signal found in the range of 1100–1200 cm^−1^, associated with the bending vibrations of C-C-N bonds, as well as the stretching vibrations of C-N bonds. In addition, the influence of Al^3+^ ions was observed in the maximum values of absorption, where displacements at longer wavelengths by about 10 units were identified: Aβ_(9–16)_ from 1134 cm^−1^ to 1146 cm^−1^, Aβ_(9–16)_G_1_^10^ from 1134 cm^−1^ to 1144 cm^−1^, Aβ_(9–16)_G_2_^13,14^ from 1135 cm^−1^ to 1148 cm^−1^, and Aβ_(9–16)_G_3_^10,13,14^ from 1133 cm^−1^ to 1148 cm^−1^. A strong influence was also noticed in the region of 1400–1450 cm^−1^, where the signal corresponding to the asymmetric stretching vibrations of the CH_3_ group, of the deformation in the plane of the CH_2_ group, and of the stretching of the CN bond underwent a partial division that led to the appearance of several peaks.

In the interval of 1500–1700 cm^−1^, specific to the amide I and amide II bonds, the aluminium ions determined a partial overlap of the two absorption maxima. Specifically, the maximum values recorded in the spectra of the Aβ_(9–16)_ peptides (1538 cm^−1^) in the presence of metal ions were attributed to disordered structures, the influence of β-conformation folding observed in the spectra of free peptides being much diminished. Similar changes were observed in the case of glycine-enriched peptides (Aβ_(9–16)_G_1_^10^, Aβ_(9–16)_G_2_^13,14^, and Aβ_(9–16)_G_3_^10,13,14^), where the presence of aluminium favoured the adoption of rather disordered structures, but α-helix and β-sheet conformers may also have been present. Nevertheless, it was difficult to see the presence of all types of conformers among the small peptide molecules from the FT-IR spectra.

Structural changes were also identified in the amide I domain, where the signal displayed in the spectra of peptides incubated with Al^3+^ ions showed a single maximum value, which suggested a β-folded structure (1631 cm^−1^, 1634 cm^−1^, and 1637 cm^−1^), unlike the spectra of free peptides, which suggested mainly α-helical conformations (Aβ_(9–16)_G_2_^13,14^ and Aβ_(9–16)_G_3_^10,13,14^).

**Figure 9:**
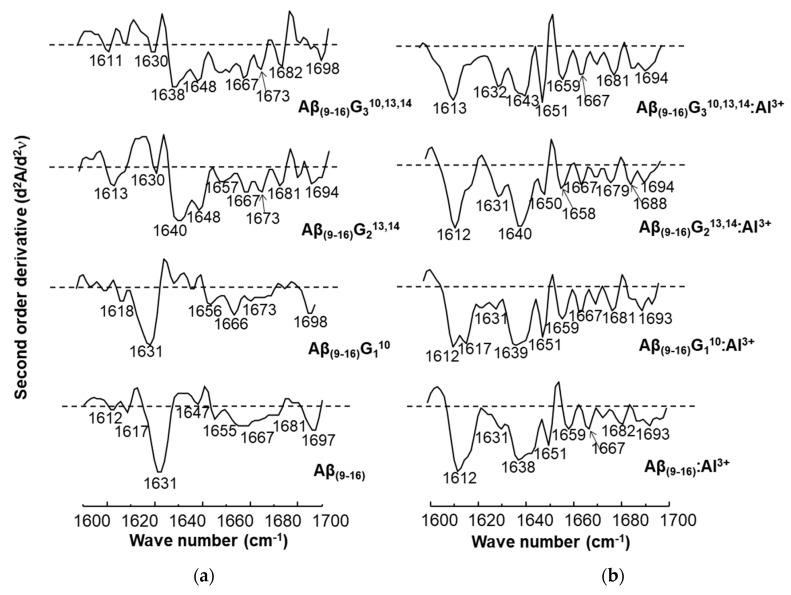
Second-order derivatives of the FT-IR spectra of amyloid peptides calculated in the range 1600–1700 cm^−1^: (**a**) in the absence of aluminium ions; (**b**) in the presence of aluminium ions.

Figure 9 shows the spectra of second-order derivatives of amyloid peptides in the absence and presence of aluminium ions. The calculation of the derivative made it possible to obtain more accurate information in the amide I region on the conformations adopted by the peptides. Although the β-folded structure of the peptides Aβ_(9–16)_ and Aβ_(9–16)_G_1_^10^ was also present when they were treated with AlCl_3_, by generating signals in the 1639–1630 cm^−1^ region, the most important result was the intense bands with maxima at 1612–1613 cm^−1^, attributed to an aggregate-type structure (Figure 9b). However, the α-helical conformation was evidenced in the structure of the peptides Aβ_(9–16)_G_2_^13,14^ and Aβ_(9–16)_G_3_^10,13,14^ in samples incubated with Al ions (1650 cm^−1^ and 1651 cm^−1^).

### 2.5. Aβ_1–16_ Conformational Changes Induced by Aluminium Ions

#### 2.5.1. Atomic Force Microscopy

**Figure 10:**
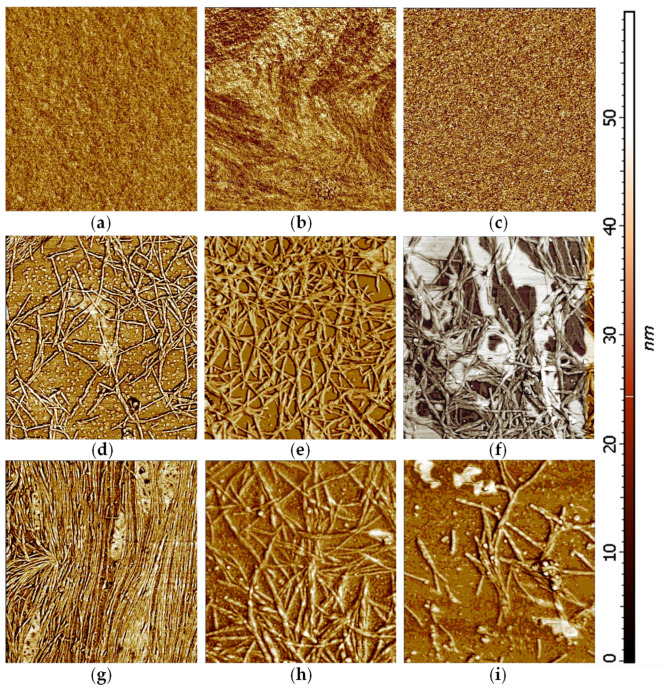
Atomic force microscopy (AFM) images of film surfaces of peptide complexes with aluminium ions. (**a**) Free Aβ_(1–16)_; (**b**) Aβ_(1–16)_ + Al_2_(SO_4_)_3_, 1:1 molar ratio; (**c**) Aβ_(1–16)_ + Al_2_(SO_4_)_3_, 1:2 molar ratio; (**d**) free Aβ_(1–16)_A_3_^6,13,14^; (**e**) Aβ_(1–16)_A_3_^6,13,14^ + Al_2_(SO_4_)_3_, 1:1 molar ratio; (**f**) Aβ_(1–16)_A_3_^6,13,14^ + Al_2_(SO_4_)_3_, 1:2 molar ratio; (**g**) free Aβ_(1–16)_S_3_^6,13,14^; (**h**) Aβ_(1–16)_S_3_^6,13,14^ + Al_2_(SO_4_)_3_, 1:1 molar ratio; (**i**) Aβ_(1–16)_S_3_^6,13,14^ + Al_2_(SO_4_)_3_, 1:2 molar ratio (peptide concentration: 256 μM; image size: 2 × 2 μm).

AFM images showed that peptide assemblies have different two-dimensional layer-shaped structures (Figure 10). A solution of free Aβ_(1–16)_ peptides produced a smooth film and no fibrils or aggregates were evident on the glass plate of the microscope. We assumed that its low tendency to aggregation is related to the hydrophilic N-terminus sequence of amyloid-β peptides. On the contrary, the alanine-rich Aβ_(1–16)_ peptide formed fibrils, most probably due to its hydrophobic characteristic, while serine-rich peptides had a pronounced tendency to fibrillation due to hydrogen bonding of serine residues. Thus, by replacing the histidine residues with alanine in the native Aβ_(1–16)_ sequence, a texture composed of differently sized fibrils appeared. Some fibrils were about 80–100 nm thick and more than 0.5 μm long, whereas the other ones were smaller but also in the nanometre range. We supposed that alanine residues highly increased the peptide hydrophobicity, as well as its α-helical proportion, thus making possible the association of molecules through non-covalent interactions. Serine-rich Aβ_(1–16)_ peptides generated even larger fibrils and aggregates. This phenomenon could be possible due to the intermolecular hydrogen bonds.

In brief, AFM images, as well as CD and FT-IR spectra, showed that the Aβ_(1–16)_ peptide conformation and fibrillation depend on the peptide sequence and the presence of aluminium in solution. The film surface of the native peptide Aβ_(1–16)_ changed only slightly with the addition of aluminum at pH 6.6 (Figure 9b). Aluminium ions induced the aggregation and fibrillation of modified peptides as demonstrated by AFM and SEM experiments. Thus, Aβ_(1–16)_A_3_^6,13,14^ generated fibrils even in the absence of Al, being a hydrophobic peptide. In the presence of Al sulphate, the fibrils became longer and thicker. The excess of aluminium salt can be observed in the AFM images for the 1:2 peptide:Al ratio. On the contrary, serine-rich peptides can form fibrils due to the hydrogen bonds.

#### 2.5.2. Scanning Electron Microscopy

**Figure 11:**
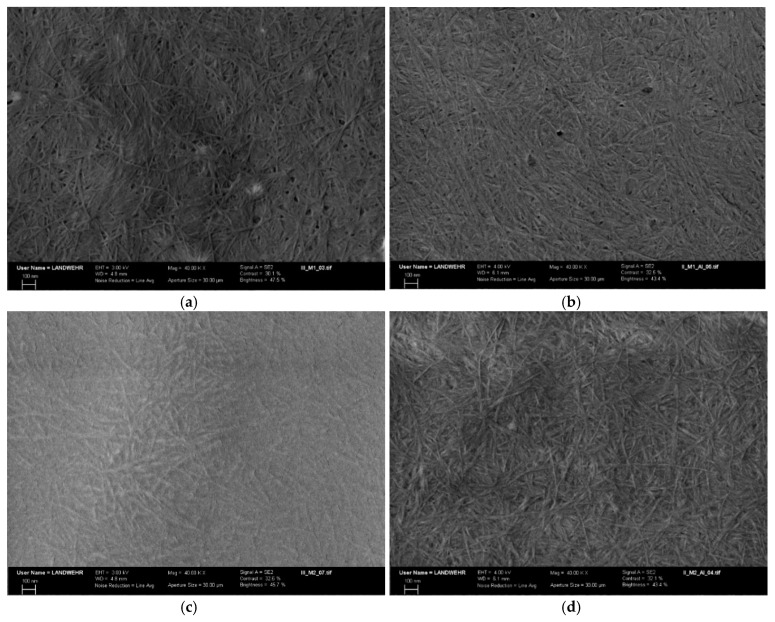
SEM images of (**a**) Aβ_(1–16)_A_3_^6,13,14^; (**b**) Aβ_(1–16)_A_3_^6,13,14^ + Al^3+^; (**c**) Aβ_(1–16)_S_3_^6,13,14^; (**d**) Aβ_(1–16)_S_3_^6,13,14^ + Al^3+^ (mag: 40 KX, aperture size: 30 µm).

The interaction of the two modified Aβ_(1–16)_ peptides with aluminium was confirmed by SEM (Figure 11) that showed a rough surface, with fibrils differently and densely distributed. These are genuine indicators of the strong interaction between the peptides and Al. SEM images of free Aβ_(1–16)_A_3_^6,13,14^ revealed numerous amyloid fibrils displaying the characteristic twist found in Aβ fibrils (Figure 11a). The modified Aβ peptides formed aggregates characterised by short fibrils which intertwine and appear rigid. With the addition of aluminium ions, the fibrils became more ordered and longer, whereas some of them seem to be finer (Figure 11b). A similar phenomenon seems to occur in the case of the formation of Aβ_(1–16)_A_3_^6,13,14^ films in the presence of aluminium ions (Figure 10e,f). As the concentration of metal ions increased, the large fibrils formed (Figure 10e), became longer, and some of them became thinner (Figure 10f). We assumed that Al increased the hydrophobicity of Aβ_(1–16)_A_3_^6,13,14^, which led to the formation of thicker and longer fibrils, followed by their division as aluminium sulphate concentration increased. In addition, these fibrils are longer and intertwined. The serine-rich peptides show short, thin fibres, which thickened and lengthened in the presence of aluminium ions (Figure 11c,d).

### 2.6. Nuclear Magnetic Resonance Spectroscopy

In the NMR study of the Aβ_(1–16)_ and Aβ_(9–16)_ fragments as well as their analogues, presented here, the interactions of these peptides with aluminium were investigated. Although we found significant changes of ^1^H-NMR spectra in the presence of aluminium sulphate, we investigate here only the spectra of some very small peptides as models. We were interested in showing that the amino acid residues may be capable of binding to or interacting with aluminium ions in solutions. However, we present here, in Figure 14, some spectra of Aβ_(9–16)_ peptide fragments in deuterium oxide solution, alone, or in the presence of aluminium ions.

**Figure 12:**
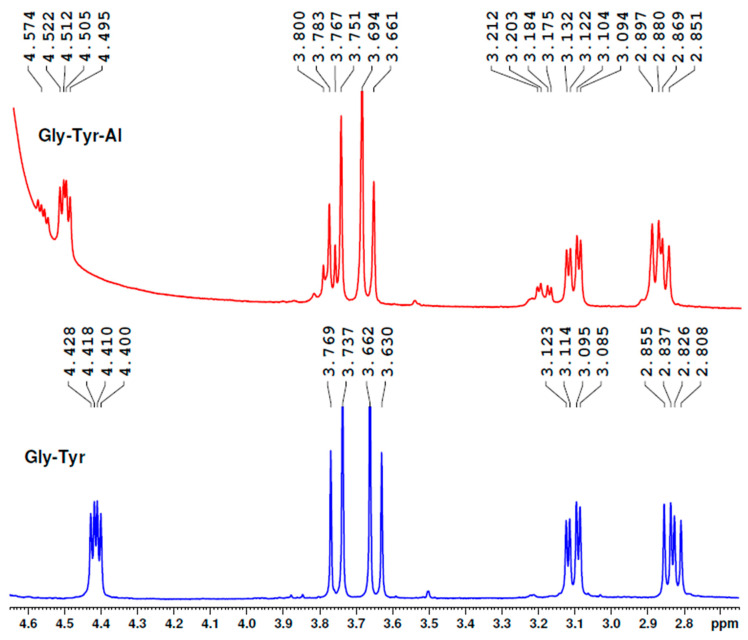
^1^H-NMR spectra of glycyl-tyrosine peptide, which demonstrate the influence of Al on the two protons of the tyrosine CH_2_ group (3.085–3.123 ppm) and the two protons of the glycine CH_2_ group (3.630–3.769 ppm).

Our experiment showed that glycyl-tyrosine bound aluminium ions in the aluminium sulphate aqueous solutions at the peptide bond region and not at the phenolic OH group (Figure 12). Although all the protons were affected by Al, the most influenced were the protons of the tyrosine CH_2_ group (the peaks shifted from 3.085–3.123 ppm to 3.125–3.212 ppm, also becoming enlarged) and the protons of the glycine CH_2_ group (from 3.630–3.769 ppm to 3.661–3.800 ppm). Of course, the proton near the COOH group was also much affected.

**Figure 13:**
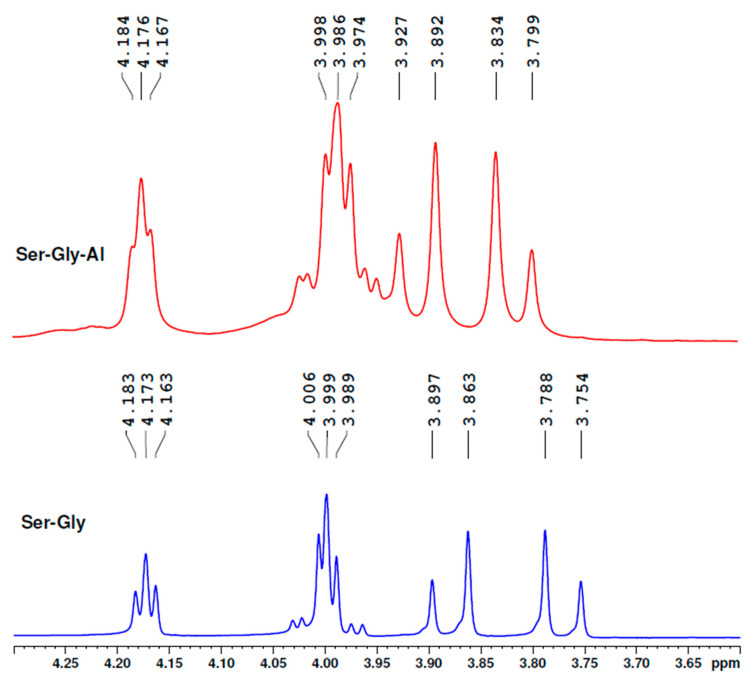
^1^H-NMR spectra of seryl-glycine peptide in the presence of aluminium sulphate demonstrate that Al ions bound to the hydroxyl group of serine.

The ^1^H-NMR spectrum of seryl-glycine contains the serine CH_2_ protons in the 3.754–3.897 ppm region (Figure 13). Under the influence of Al, we found the peaks shifted to 3.799–3.927 ppm, but the distance between the highest signals was smaller. These findings demonstrate that aluminium ions are bound to serine OH groups. In addition, the two protons of the glycine CH_2_ group were found to be shifted toward higher ppm values and wider. Besides, the CH groups of serine residues bound to amino groups were also modified.

These data confirm that the peptide backbone is also involved in aluminium binding and the hydroxyl group of serine also binds aluminium ions.

**Figure 14:**
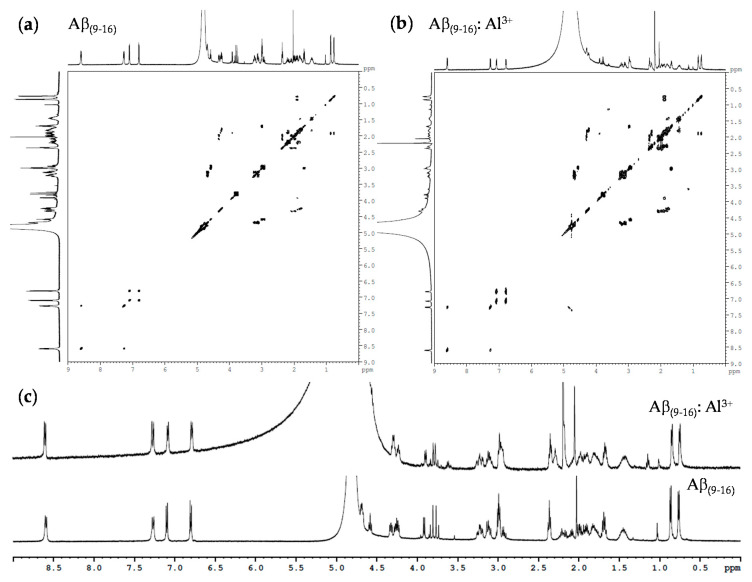
^1^H-NMR spectra of Aβ_(9–16)_ peptide in the presence of aluminium sulphate: (**a**) COSY ^1^H NMR spectrum of Aβ_(9–16)_ peptide; (**b**) COSY ^1^H NMR spectrum of Aβ_(9–16)_ in the presence of Al^3+^; (**c**) ^1^H-NMR spectra.

Figure 14 shows that the NMR spectra of the Aβ_(9–16)_ peptide is slightly changed in the presence of aluminium ions. However, we also studied by NMR spectroscopy Al binding to Aβ_(9–16)_, Aβ_(1–16)_, or only its interaction with these peptides, as well as their modified variants, and continued with Aβ_(1–40)_ and its variants in which the histidine residues were replaced with serine and alanine ones (not shown). Some other experiments are in progress, in which larger amounts of peptides are used, and, therefore, a new manuscript is expected to disseminate the results.

## 3. Discussion

Interaction of metal ions with Aβ is mediated mostly by the N-terminal Aβ_(1−16)_ domain and appears to play an important role in AD progression [36]. Although Al ions also were reported to bind to some residues found in the Aβ_(1–16)_ sequence, no measurable amounts of Aβ_(1–28)_-Al^3+^ adducts were noticed by other authors within their experiments by ^1^H NMR and electrospray ionisation mass spectrometry (ESI-MS) at physiological pH [37]. These were the incentive conditions for the present investigation of Al interactions with Aβ in which we synthesised several variants of the Aβ_(1–16)_ and Aβ_(9–16)_ peptides, and which contain histidine residues replaced by alanine, serine, or glycine. Since the interaction of amyloid-β peptides with Al ions might be involved in AD pathogenesis, we investigated aluminium binding to the newly synthesised peptides by MS, FT-IR, CD, AFM, and NMR spectroscopy.

Coordination of Al ions to the *N*-terminus of Aβ at the acidic residues Glu^3^, Asp^7^, and Glu^11^ induces significant helical content, which means a major impact on the structure and dynamics of the peptide and reducing the impact of salt bridges, as well as the flexibility of binding residues and increasing that of terminal residues [23]. High helical content and disruption of salt bridges lead to a characteristic tertiary structure, as shown by heat maps of contact between residues, as well as representative clusters of trajectories [23]. The Al-induced flexibility allows for significant quantities of helical secondary structure to develop. Indeed, such metal ions affect residues 11–20 and 26–36 [23]. Salt bridges are strongly affected by the presence of the Al ion in the 11–16 region, as we demonstrated here. This, along with hydrogen bond patterns of serine-rich peptides that reflect the rather high helical content, lead to characteristic patterns in the tertiary structure in which stable contacts between salt-bridged pairs, as well as residues bound to Al ions, are apparent.

Therefore, FT-IR and CD are convenient methods to predict metal binding and conformational changes.

Although AD is not curable, some treatments are available to slow down the disease progression and reduce the cognitive impairment and behavioural problems, but do not stop the progression of neurodegeneration [38]. Our research may show new details about Aβ aggregation and fibrillation associated with AD and highlight the role of aluminium ions in creating a hydrophobic environment that increases the tendency of Aβ peptides to form plaques in AD brains.

The presented results are in good agreement with recent data demonstrating that the topology of Aβ_(1–16)_ chelated with the Al^3+^ ion remains preserved, as compared to the free peptide [39], while another study performed with the Aβ_(1–28)_ fragment revealed no measurable amounts of Al–peptide adducts upon peptide–metal mixing, regardless of experimental conditions [37]. We also considered that histidine residues are not involved in Al binding, but they may influence aluminium binding due to the rigidity of the peptide backbone.

**Figure 15:**
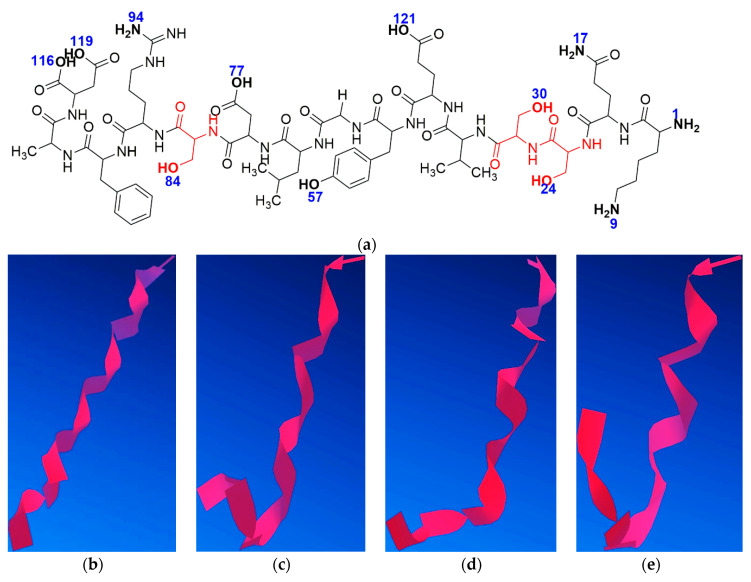

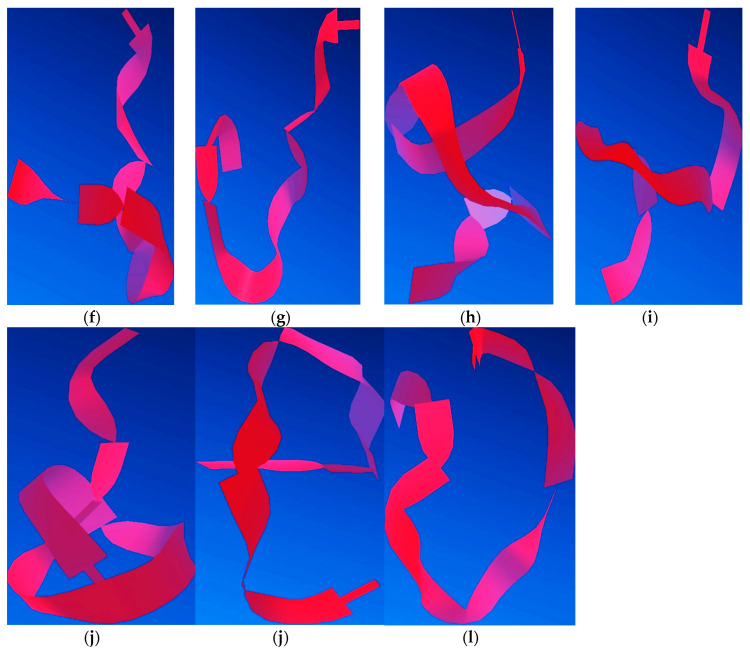
(**a**) Aβ_(1–16)_S_3_^6,13,14^ sequence with the numbered atoms involved in the bonding process; (**b**) Aβ_(1–16)_S_3_^6,13,14^; (**c**) Aβ_(1–16)_S_3_^6,13,14^(24.30)9; (**d**) Aβ_(1–16)_S_3_^6,13,14^(24.30)1; (**e**) Aβ_(1–16)_S_3_^6,13,14^(24.30)17; (**f**) Aβ_(1–16)_S_3_^6,13,14^(24.30)121; (**g**) Aβ_(1–16)_S_3_^6,13,14^(24.30)57; (**h**) Aβ_(1–16)_S_3_^6,13,14^(24.30)77; (**i**) Aβ_(1–16)_S_3_^6,13,14^(24.30)84; (**j**) Aβ_(1–16)_S_3_^6,13,14^(24.30)116; (**k**) Aβ_(1–16)_S_3_^6,13,14^(24.30)119; (**l**) Aβ_(1–16)_S_3_^6,13,14^(24.30)94.

The process of minimising peptide energy in silico was performed involving steric energy (Figure 15). For this study, the modified peptide Aβ_(1–16)_S_3_^6,13,14^ was taken into consideration to understand the influence of the Al^3+^ ion on its secondary structure. Because serine residues introduced into the native Aβ_(1–16)_ due to the hydroxyl group can form specific bonds with aluminium ions, we analysed the influence of these functional groups in complementarity with other amino and hydroxyl functions in the Aβ_(1–16)_ secondary structure. We assumed that the two oxygen atoms of the hydroxyl groups of the two serine residues at positions 13 and 14 (atoms no. 24 and 30) bind to the Al^3+^ ion, while a third bond is formed between Al and the atom of oxygen or nitrogen from the amino and hydroxyl functional groups (numbered according to Figure 15a). Thus, we found 11 possible variants of such an interaction with Al. Each version was applied to the process of minimising energy in order to obtain the most stable conformation from the energy point of view.

**Figure 16:**
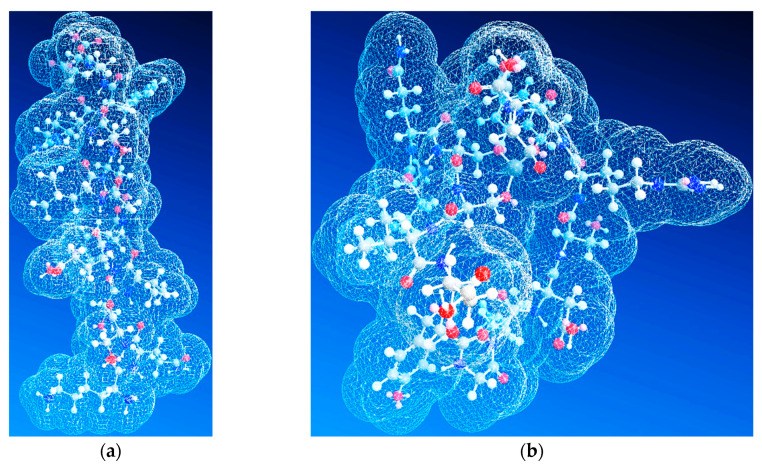
Accessibility of solvents to: (**a**) Aβ_(1–16)_S_3_^6,13,14^; (**b**) Aβ_(1–16)_S_3_^6,13,14^(24.30)94 + Al^3+^.

Molecular modelling indicated that the site of aluminium binding migrates toward the C-terminus of the peptide, and the peptide backbone tends to adopt a rather β-sheet conformation, thus reducing the proportion of α-helix conformers that induced the specific elasticity of the peptide. Therefore, these findings suggest that with the interaction of aluminium ions with nitrogen and oxygen atoms, the rigidity of the structure increases, thus giving the peptide an attribute of inaccessibility to solvents, according to Figure 16. This may also suggest an increase in the hydrophobicity of the serine-rich peptide, which induces an increased tendency to aggregate. Nevertheless, we should keep in mind that a large proportion of Aβ molecules are free and that a large amount of Al is unbound, acting in the form of inorganic compounds. These theoretical results confirm experimental CD, AFM, and FT-IR data.

## 4. Materials and Methods

All chemicals were of analytical or higher grade and purchased from well-recognised commercial sources. The solvents for peptide synthesis were commercial analytical grade and were redistilled before use. All solutions and buffers were prepared using MilliQ-grade water (18.2 MΩ∙cm) from a Millipore water purification system (Milford, MA, USA). As solid support for the peptide synthesis, a Rink amide resin procured from Sigma-Aldrich Chemie GmbH was used (Taufkirchen, Germany). The following side chain-protected amino acids were used: Fmoc-Asp(OtBu)-OH, Fmoc-Ala-OH, Fmoc-Glu(OtBu)-OH, Fmoc-Phe-OH, Fmoc-Arg(Pbf)-OH, Fmoc-l-His(Trt)-OH, Fmoc-Ser(tBu)-OH, Fmoc-Gly-OH, Fmoc-l-Tyr(tBu)-OH, Fmoc-Val-OH, Fmoc-l-Gln(Trt)-OH, and Fmoc-Lys(Boc)-OH. Benzotriazol-1-yl-oxytripyrrolidino-phosphonium hexafluorophosphate (PyBOP) was used as an activator and purchased from Novabiochem (Darmstadt, Germany). Triisopropylsilan (TIS), dichloromethane (DCM), 4-methylmorpholine (NMM), piperidine, trifluoroacetic acid, and α-cyano-4-hydroxycinnamic acid (CHCA) were obtained from Sigma-Aldrich Ltd. (St. Louis, MO, USA). *N*,*N*-dimethylformamide (DMF) was purchased from Carl Roth (Karlsruhe, Germany), while ethanol and diethyl ether were achieved from Scharlab (Barcelona, Spain). Acetonitrile (ACN) was bought from Merck (Darmstadt, Germany). Deuterium oxide (D_2_O) was supplied by Cambridge Isotope Laboratories, Inc. (Tewksbury, MA, USA), whereas aluminium sulphate and aluminium chloride were from Merck KGaA (Darmstadt, Germany). Piperidine (PYP) and bromophenol blue were obtained from Merck (Germany), while bradykinin, substance P, renin, adrenocorticotropin (ACTH), and oxidized insulin B-chain were from Sigma, city, Germany.

In addition to the peptides belonging to the Aβ_(1–16)_ and Aβ_(9–16)_ group, which were synthesised in our laboratory, other peptides and an amino acid were associated with the experiments of aluminium binding. The dipeptide glycyl-tyrosine (Gly-Tyr) was supplied by Fluka Chem. Co. (Steinheim, Germany) and chosen as a model for the ^9^GY^10^ peptide fragment in the binding of aluminium ions. In addition, another dipeptide, seryl-glycine (Sigma), was used to study the behaviour of the system ^8^SG^9^. Because the C-terminus of these two peptides is not amidated or protected, we also introduced in our experiments tyrosinamide (Tyr-NH_2_) obtained from Sigma-Aldrich.

### 4.1. Synthesis of Aβ Peptide Fragments

More detailed description of solid phase peptide synthesis was provided in our previous papers [30,32]. The sequence of Aβ_(1–16)_ and those of the newly synthesised peptides were: Aβ_(1–16)_:H-^1^DAEFR**H**DSGYEV**HH**QK^16^-NH_2_, Aβ_(1–16)_A_3_^6,13,14^_:_H-^1^DAEFR**A**DSGYEV**AA**QK^16^-NH_2_, and Aβ_(1–16)_S_3_^6,13,14^:H-^1^DAEFR**S**DSGYEV**SS**QK^16^-NH_2_. The peptides were prepared by solid-phase peptide synthesis (SPPS) on a Fmoc Rink amide MBHA resin (MBHA, 4-methyl-benzihidril-amine; 0.51 mmol g^−1^) according to Fmoc/tBu chemistry, using an automated ResPepSL Peptide Synthesizer from Intavis (City, Germany). The synthesis protocol was as follows: (1) DMF washing, (2) Fmoc cleavage with 20% piperidine in DMF, (3) DMF washing, (4) coupling of Fmoc amino acid:PyBOP:NMM in DMF, and (5) DMF washing. Following the last amino acid loading to the chain, the Fmoc protecting group was removed and the peptide with a free amino terminal was washed with ethanol and DCM and subsequently subjected to lyophilisation until the next day. For the lyophilisation of peptides, a lyophiliser from Martin Christ Alpha 1-2 LDplus (Martin Christ, Osterode am Harz, Germany) was used. The peptides were cleaved from the resins at room temperature using a cleavage mixture consisting of 95% trifluoroacetic acid (TFA) as the cleavage agent, 2.5% triisopropylsilan, and 2.5% deionised water for 3 h. After cleavage, the peptides were precipitated with cold (−20 °C) diethyl-ether overnight, then subjected to simple filtration. The solid material was dissolved in 5% aqueous solution of acetic acid prior to dry freezing.

The Aβ_(9–16)_ peptide (H-^9^GYEVHHQK^16^-NH_2_) and the modified peptides Aβ_(9–16)_G_1_^10^ (H-^9^G**G**EVHHQK^16^-NH_2_), Aβ_(9–16)_G_2_^13,14^ (H-^9^GYEV**GG**QK^16^-NH_2_), and Aβ_(9–16)_G_3_^10,13,14^ (H-^9^GGEVGGQK^16^-NH_2_) were manually synthesised by solid-phase peptide synthesis (SPPS) on a Fmoc Rink amide MBHA resin (0.48 mmol/g) according to Fmoc/tBu chemistry in a plastic syringe [40,41].

### 4.2. Reversed-Phase High-Performance Liquid Chromatography

Aβ_(1–16)_ peptides were purified by an RP-HPLC SpectraSystem (Thermo Fisher Scientific, Dreieich, Germany) on a semi-preparative Vydac C8 column (250 mm × 10 mm, 10 µm silica, and 300 Å pore size) from Grace Columns. The mobile phase was a mixture of eluent A (0.1% TFA in water) and eluent B (0.1% TFA in acetonitrile–water (80:20, *v*/*v*)). A linear gradient elution was used for Aβ separation (0 min 5% B; 5 min 5% B; 60 min 60% B; 65 min 100% B). A flow rate of 1.5 mL min^−1^ was used and the peptides were detected at 220 nm.

Analytical RP-HPLC was also performed on a SpectraSystem using an analytical Vydac C8 column (250 mm × 4.6 mm) with 5 µm silica (300 Å pore size) as a stationary phase (Grace Columns). The method was run at 1.0 mL min^−1^ using a mobile phase similar to the semi-preparative method.

Purification of Aβ_(9–16)_ peptides was done by RP-HPLC on a UHPLC Dionex UltiMate 3000 instrument (Thermo Scientific, Bremen, Germany) on a Vydac C18 column (250 mm × 4.6 mm), from Grace Columns, using two concentration gradients.

### 4.3. MALDI-ToF Mass Spectrometry

Matrix-assisted laser desorption/ionisation time-of-flight mass spectrometry (MALDI-ToF MS) analysis was carried out with a Bruker Ultraflex MALDI ToF/ToF mass spectrometer operated in positive reflectron mode and equipped with a pulsed nitrogen UV laser (Bruker Daltonics, Hamburg, Germany). For this analysis, the samples were co-crystallised with an excess of organic matrix capable of absorbing at 337 nm and volatilising under the action of laser radiation. As a matrix, a saturated solution of α-cyano-4-hydroxy-cinnamic acid (CHCA) dissolved in a solution containing 2:1 ACN:0.1% TFA in MilliQ was used for peptide mapping. Using the dried drop method, which implies adding first 1 µL of sample and over it 1 µL of freshly prepared matrix solution, the mixture was deposited on a conductive metallic plate called a target and allowed to dry. After co-crystallisation, the metal plate was introduced into the mass spectrometer and bombarded with short laser pulses. The desorbed and ionised molecules were accelerated by an electrostatic field and discharged through a high-fly metal flight tube. Depending on their mass, ionised molecules reached the detector at different times.

The spectra were recorded in positive reflectron mode using the following parameters: 20 kV acceleration voltage, 40% grid voltage, 140 ns delay, low-mass gate of 500 Da, and an acquisition mass range of 600-3500 Da. The final mass spectrum represented an accumulation of 300 shots per acquisition. The obtained spectra were processed using Bruker’s Flex Analysis 3.4 software.

### 4.4. ESI Type Mass Spectrometry

Electrospray ionisation ion trap mass spectra (ESI-MS) were recorded in positive mode by scanning from *m*/*z* 200 to *m*/*z* 2000 on an Esquire 3000+ ion trap mass spectrometer (Bruker Daltonik, Bremen, Germany). Each spectrum, corrected for the blank baseline, was the average of 15 scans. The ion source parameters were as follows: 19 psi nebulising gas (nitrogen), 9 L∙min^–1^ of drying gas (nitrogen) at a dry temperature of 300 °C, capillary voltage 4000, skimmer –4V, and capillary exit 98.50 V. The flow rate of the sample was 600 μL h^−1^. The collision-induced dissociation technique (CID) generated fragmentation of paternal ions. Collision-induced dissociation was done using argon as the target gas.

### 4.5. Peptide–Metal Complex Preparation

*Al-Aβ_(9–16)_ peptide complex*. A stock 384 μM solution of Aβ_(9–16)_ peptide and another one of 1.536 mM aluminium sulphate in ammonium acetate buffer (pH = 7.4) were prepared. To obtain 1:1 molar ratio solutions, 100 μL of 384 μM Aβ_(1–16)_ solution, 25 μL solution of 1.536 mM metal ions and 25 μL buffer solution were mixed in an Eppendorf vial. The peptide concentration in the final solution was 256 μM.

*Al-Aβ_(1–16)_ peptide complexes*. AFM and SEM studies were done using the native Aβ_(1–16)_ peptide and its variants with alanine and serine, Aβ_(1–16)_A_3_^6,13,14^, and Aβ_(1–16)_S_3_^6,13,14^ at a concentration of 256 μM, treated with aluminium sulphate (1:1 and 1:2 molar ratios) for 24 h at 25°C.

*Al-Aβ_(9–16)_ peptide complexes*. Separately, the newly synthesised, glycine-rich Aβ_(9–16)_ peptides were subjected to interaction with aluminium ions in aqueous AlCl_3_ solutions in order to compare their affinity to Al with that of native Aβ_(9–16)_. Here, solutions of each Aβ_(9–16)_ peptide (8 mM) were mixed at pH 6.6 with aluminium chloride to obtain a peptide:metal ion molar ratio of 1:10. The resulting solutions were incubated (Thermomixer Compact Eppendorf AG 22331, City, Germany) for 20 h at 24 °C and 350 rpm. Finally, the samples were lyophilised and subjected to MS and FT-IR.

### 4.6. Circular Dichroism Spectroscopy (CD)

CD spectra were recorded on a Jasco J-720 spectropolarimeter (JASCO, Espoo, Finland) at room temperature in a 0.5 mm quartz cell under a constant stream of nitrogen (4 L/min). Buffer with ammonium acetate (15 mM) of pH 6.6 or pH 7.4 was used as the solvent. The Aβ_(1–16)_ peptide concentration was 256 µM. Spectra were the average of five scans between λ 180 and 260 nm. The results were expressed as molar ellipticity, after subtracting the buffer spectrum. A HANNA pH 211 microprocessor pH meter was used to measure the pH values.

### 4.7. Atomic Force Microscopy

In-phase and in-height AFM images were taken at room temperature (22 °C) on a SPM Solver PRO-M AFM (NT-MTD Co. Zelenograd, Moscow, Russia) using the tapping mode. All images were acquired using a high-resolution noncontact “Golden” silicon NSG10/Au/50 cantilever with Au conductive coating. The cantilever was 100 µm in length, 35 µm in width, 2 µm thick, and had a typical tip radius of 10 nm. A resonant frequency of 254 kHz and a force constant of 11.5 N m^−1^ were applied. All AFM images were obtained at a resolution of 256 × 256 pixels on a scale of 2 µm × 2 µm. We used AFM to characterise the film surface of native Aβ_(1–16)_ peptides in comparison with that of Aβ_(1–16)_A_3_^6,13,14^ and Aβ_(1–16)_S_3_^6,13,14^ upon incubation with aluminium at a concentration of 256 μM (1:1 and 1:2 molar ratios) for 24 h at 25°C. To examine the topography of aggregates, two to three drops of each sample (approximately 50 µL of sample solution) were allowed to dry overnight on small glass slides in dust-free medium (covered with a Petri dish), at room temperature.

### 4.8. Scanning Electron Microscopy

A scanning electron microscope (SEM; Ultra plus Scanning Electron Microscope from Carl Zeiss NTS) (Manufacturer, City, State abbr. if USA, Country), operating at 4 kV with secondary electrons, in high-vacuum mode, was used to observe the morphological properties of the Al–peptide complexes. The SEM studies were performed on samples dried on small glass slides, fixed on copper supports using carbon tape, and covered with a thin layer of platinum to avoid electrostatic charging. We used SEM to characterise the fibril morphology of aggregates formed by Aβ(1–16)A_3_^6,13,14^ and Aβ(1–16)S_3_^6,13,14^ upon incubation with aluminium sulphate (peptide concentration 256 μM; 1:1 molar ratio) for 24 h at 25 °C.

### 4.9. Fourier Transform Infrared Spectroscopy

The infrared spectra were measured using a Shimadzu 8400S FT-IR spectrophotometer (Shimadzu, Japan). The FT-IR spectra were recorded from 4000 to 400 cm^−1^ in spectroscopic-grade CsI with a detector at 2 cm^−1^ resolution and 20 scans per sample working in transmission mode. The collected FT-IR spectra were compared with the standard spectra of the functional groups.

### 4.10. NMR Spectroscopy

Proton NMR data were recorded by Dr. Catalina Ionica Ciobanu on a Bruker Avance III, 500 MHz frequency spectrometer, equipped with a 5 mm PABBO detection probe and operating at 500.19 MHz for ^1^H nucleus. Chemical shifts are reported in δ units (ppm), relative to the solvent residual peak (D_2_O, ^1^H:4.79 ppm). Coupling constants are reported in Hertz (Hz). The number of scans for ^1^H-NMR experiments was 256 and four scans for two-dimensional ^1^H-^1^H NMR spectra. The peptide sample was prepared in D_2_O as a solvent. The experiment was recorded without the introduction of a presaturation pulse for solvent signal suppression.

### 4.11. Data Analysis

The mass spectrometric data analyses were performed using the software Bruker Daltonics DataAnalysis 3.3. The molecular weight determination of peptides, as well as the prediction of the corresponding fragments in the MS/MS process of peptides and Al-fragment adducts, were conducted using GPMAW 6.11 software (General Protein/Mass Analysis for Windows, Lighthouse Data, Odense M, Denmark) [42]. The monoisotopic peak list was compared against data calculated by GPMAW. AFM image analysis was done with scanning probe microscopy software, WSxM 4.0 Develop 10.0 (NT-MTD Co. Zelenograd, Moscow, Russia). The obtained data following IR spectroscopy were processed using Origin software. Computer simulation was performed with the help of the Chem3D Ultra 10.0 program.

## 5. Conclusions

Our findings suggest that the *N*-terminal 1–16 sequence of Aβ peptides has the is involved in aluminium ion binding associated with AD. The key observation in our study is that aluminium ions interact with the N-terminus Aβ_(1–16)_ sequence of amyloid-β peptides. In addition, the Aβ_(9–16)_ peptide fragment also interacts with Al, as the FT-IR experiments suggest. Consequently, several variants of Aβ_(1–16)_ and Aβ_(9–16)_ peptide fragments were synthesised and used to study their interaction with aluminium ions. These ions were suspected to induce the formation of Aβ fibrils and aggregates. Through the formation of metal ion adducts during the MALDI-ToF MS measurements, we used MS/MS spectra to investigate aluminium binding to amyloid-β peptide fragments and analogues, and to identify the most probable binding sites. AFM images, as well as CD and FT-IR spectra, showed that the Aβ_(1–16)_ peptide conformation and fibrillation depend on the sequence of peptides and the presence of aluminium in solution. Free Aβ_(1–16)_ peptides, which are the hydrophilic N-terminus sequences of amyloid-β peptides, generated a smooth film surface because of their low tendency to aggregation. Instead, alanine-rich Aβ_(1–16)_ formed fibrils, most probably due to its hydrophobic characteristic, while serine-rich peptides had a pronounced tendency to fibrillation, probably due to the hydrogen bonding of serine residues. Aluminium ions induce peptide aggregation and fibrillation, as demonstrated by AFM and SEM experiments. However, Al may have a higher affinity to amyloid peptides at a lower pH, and its binding could be pH dependent. Investigation of such short amyloid-β peptide fragments and analogues may provide clues for plaque formation under aggregation conditions and may facilitate the design of potential drugs for these targets. However, further research is needed to better understand the multiple interactions of aluminium ions with Aβ peptides for Alzheimer’s disease.

## Figures and Tables

**Table 1:** The *m*/*z* values calculated and measured experimentally using MS/MS spectra of ions [M+Al(III)-2H]^+^ following the collision-induced dissociation (CID) fragmentation (MALDI mass spectrometer).

Peptide	Sequence	Theoretical *(m*/*z)*	Experimental *(m*/*z)*
**Aβ**_(**9–16**)_ GYEVHHQK	H	110.0	110.1
	*b_4_*-NH_2_	433.2	433.3
	VHHQ	502.2	502.3
	*b_5_*-NH_2_	570.2	570.4
	*a_5_* + Al (*a_5_* + Al − 3H)	582.2	582.2
	*b_5_* + Al (*b_5_* + Al − 3H)	610.2	610.3
	*y_5_* + Al (*y_5_* + Al − 3H)	671.3	671.5
	*a_6_* + Al (*a_6_* + Al − 3H)	719.3	719.4
	*b_6_* + Al (*b_6_* + Al − 3H)	747.3	747.5
	*x_6_* + Al (*x_6_* + Al − 3H) *a_7_* + Al (*a_7_* + Al − 3H)	824.3 847.3	824.0 847.6
**Aβ**_(**9–16**)_**G_1_^10^** G**G**EVHHQK	H	110.0	110.2
	*c_2_*	134.0	134.2
	HQ	266.1	266.2
	*a_5_-H_2_O*	434.2	433.4
	*y_4_* + Al (*y_4_* + Al − 3H)	572.3	572.6
	*a_6_* +Al *(a_6_* + Al − 3H)	613.2	613.6
	*b_6_* +Al (*b_6_* + Al − 3H)	641.2	641.6
	*y_5_* +Al (*y_5_* + Al − 3H)	671.3	670.7
	*a_7_* +Al (*a_7_* + Al − 3H)	741.3	741.8
**Aβ**_(**9–16**)_**G_2_^13,14^** GYEV**GG**QK	*y_4_* +Al (*y_4_* + Al − 3H)	412.2	411.6
	*c_5_*-NH_2_	509.2	509.7
**Aβ**_(**9–16**)_**G_3_^10,13,14^** G**G**EV**GG**QK	VGGQ *y_5_* + Al (*y_5_* + Al − 3H)	342.1 511.2	342.3 510.7
	*z_7_*	654.3	654.7

## Abbreviations

Aβ, amyloid-β peptide; AD, Alzheimer’s disease; AFM, atomic force microscopy; Al, aluminium; CD, circular dichroism spectroscopy; FT-IR, Fourier transform infrared spectroscopy; SEM, scanning electron microscopy; SPPS, solid-phase peptide synthesis.
